# Postoperative delirium after long-term general anesthesia in elderly patients, how to reduce it? Protocol of a double-blinded, randomized, placebo-controlled trial

**DOI:** 10.1097/MD.0000000000025885

**Published:** 2021-06-04

**Authors:** Xiaoyuan Sui, Qingmei Duan, Kunling Liu, Cuicui Li

**Affiliations:** a Chongqing Shapingba District People's Hospital; b Xinqiao Community Health Service Center, Shapingba District, Chongqing, China.

**Keywords:** dexmedetomidine, postoperative delirium, long-term operation, randomized controlled trial, protocol

## Abstract

**Background:**

Long operation duration (>4 hours’ anesthesia) of laparotomy in elderly patients would increase the risk of postoperative delirium (POD), which is characterized by acute cognitive dysfunction, changes in the level of consciousness, obvious attention disorder, emotional disorder, and sleep-waking cycle disorder. The occurrence of POD is closely related to the risk of death, and it will also seriously affect the cognitive function of patients, prolong postoperative hospital stays, and increase medical expenses. It is known that dexmetomidine could function in sedation, analgesia and anti-sympathetic effect, and it also could simulate the normal sleep state of human body, but there is still a lack of clinical study of dexmedetomidine on the incidence of POD in elderly patients undergoing long-term general anesthesia in laparotomy.

**Methods:**

This is a single-center, double-blinded, randomized controlled study. With the approval of the Ethics Committee of Chongqing Shapingba District People's Hospital, participants who meet the requirements will be randomly divided into the treatment group (continuous infusion of dexmetomidine) and the control group (continuous infusion of 0.9% sodium chloride solution) in a ratio of 1:1. The incidence of delirium, cognitive function score, inflammatory factors, and adverse reactions will be evaluated after the operation. Finally, the data will be analyzed by SPSS 22.0.

**Conclusion:**

The results of this study will explore the efficacy and safety of dexmetomidine in reducing the incidence of postoperative delirium in elderly patients undergoing long-term general anesthesia in laparotomy.

**Trial registration:**

OSF Registration number: DOI 10.17605/OSF.IO/2GJY6

## 1 Introduction

Hepatobiliary surgery usually takes a longer anesthesia time for operation for its complexity and multi-steps. In addition, due to the limitations of patient quality, anatomy, and equipment, sometimes minimally invasive surgery is not available, so conventional laparotomy will be applied in many elderly patients. However, longer operation duration (>4 hours anesthesia) of laparotomy in elderly patients would increase the risk of many complications, for example, postoperative delirium (POD),^[1]^ which is characterized by acute cognitive dysfunction, changes in the level of consciousness, obvious attention disorder, emotional disorder and sleep-waking cycle disorder.^[2]^

POD commonly occurs in elderly patients,^[3]^ and research showed that the proportion of POD in the elderly was 15% to 50%, which varied by the location, time, and type of operation.^[4]^ The occurrence of POD is closely related to the risk of death, and the risk of death in patients with POD will be increased by 11% in 3 months and 17% in 1 year.^[5]^ POD will also seriously affect the cognitive function of patients,^[6,7]^ prolong postoperative hospital stays, and increase medical expenses.^[8]^ Therefore, how to reduce the incidence of postoperative delirium in elderly patients with laparotomy is an urgent issue to be solved.

Dexmedetomidine is a central alpha-2 adrenergic agonist, which is commonly used as an auxiliary sedative in clinical anesthesia. It could function in sedation, analgesia and anti-sympathetic effect, and it also could simulate the normal sleep state of human body.^[9]^ It has been reported that dexmetomidine can improve the awakening quality of patients under general anesthesia and reduce the incidence of delirium in elderly patients after cardiac surgery, colorectal cancer surgery, and joint replacement.^[10–12]^ However, there is still a lack of clinical study of dexmedetomidine on the incidence of POD in elderly patients after long-term laparotomy. Therefore, we plan to conduct this prospective randomized controlled study to explore the efficacy and safety of intraoperative use of dexmetomidine on POD in elderly patients undergoing long-term general anesthesia in laparotomy.

## 2 Methods

### 2.1 Study design

**Figure 1:**
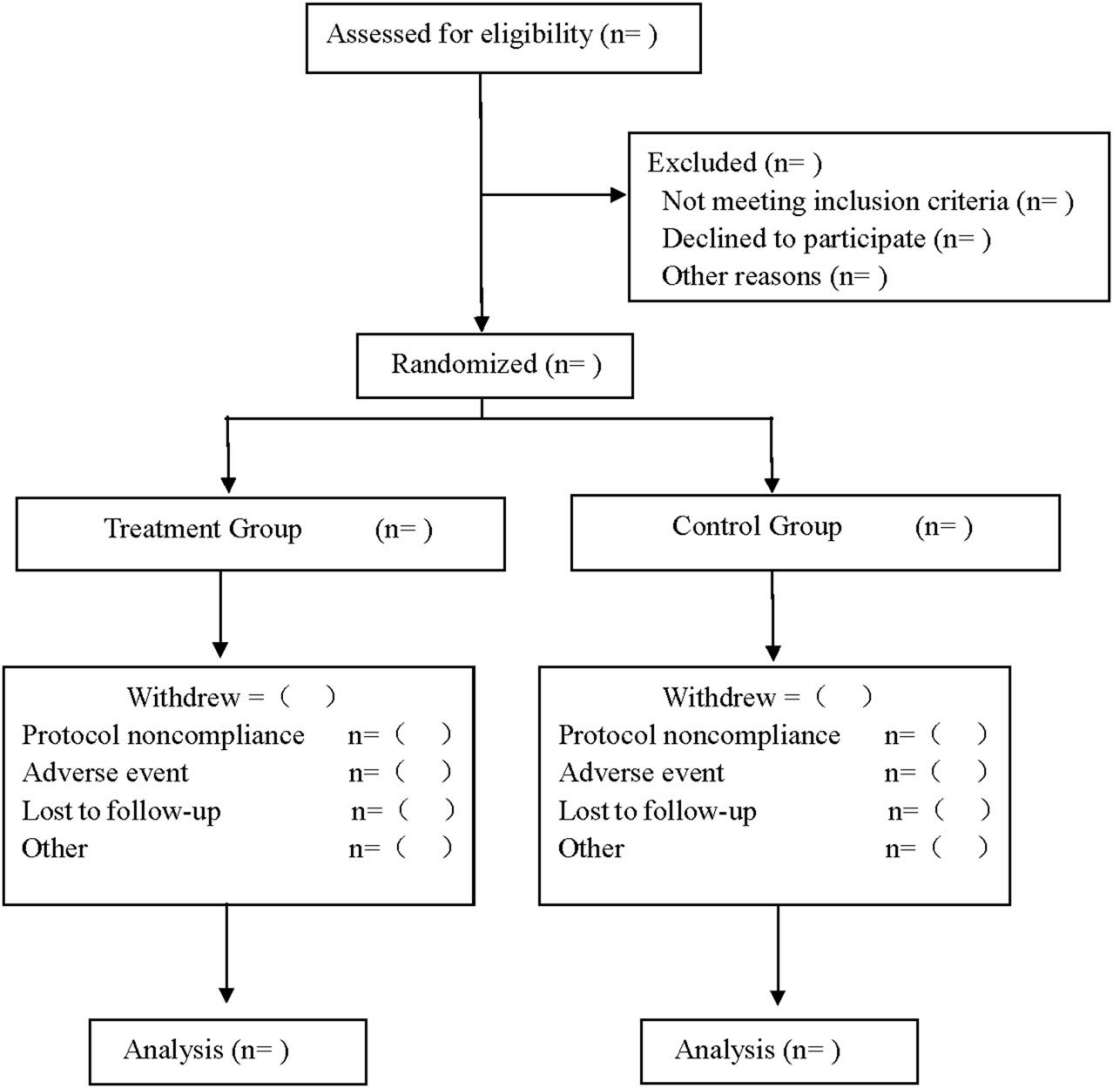
Flow diagram.

This study is a prospective, single-center, single-blind, randomized controlled clinical trial. This research program has been approved by the Ethics Committee of Chongqing Shapingba District People's Hospital and it fully complies with the Helsinki Declaration in the process of implementation. The design, implementation, and results report of this scheme will be carried out in accordance with the requirements of SPIRIT 2013 Statement^[13]^ and CONSORT 2010 Statement.^[14]^ This research program has been registered in the open scientific framework (OSF) (registration number: DOI 10.17605/OSF.IO/2GJY6). A CONSORT flow diagram is shown in Figure 1.

### 2.2 Case

#### 2.2.1 Estimation of case source and sample size

We will include patients through pre-hospital recruitment and screening of inpatients, and after qualification assessment, patients will be informed of the scheme and purpose of the trial, and they will be included in the study only after signing an informed consent form.

The sample size estimation will be based on the incidence of delirium on the first day after operation. According to the preexperimental results, the incidence rate of the treatment group is 15.5%, and 42% in the control group. Taking α = 0.05, β = 0.1, 46 samples will be needed in each group after calculated by PASS 15.0 software. A withdrawal rate of not /.20% is considered, and 58 cases are required in each group.

#### 2.2.2 Inclusion criteria

1. Patients aging 60–75 years’ old;
2. Patients receiving hepatobiliary laparotomy with an estimated duration of >4 hours in general anesthesia;
3. American Society of Anesthesiologists grade, grade I–III;
4. Patients with signed informed consent form.

#### 2.2.3 Exclusion criteria

1. Patients with a history of neuromuscular and endocrine system diseases, and mental system diseases;
2. Patients with a history of long-term use of alcohol, anti-depressant, anti-anxiety, and other neuropsychiatric drugs;
3. Patients with a history of major cardiovascular and respiratory diseases and serious damage to liver and kidney functions;
4. Patients with the score of mini-mental state examination <23 before operation;

#### 2.2.4 Discontinuation criteria

1. Patients with intraoperative blood pressure exceeding 10% of the baseline value and lasting for >10 minutes;
2. Patients with intraoperative blood loss >20% of the basic blood volume;
3. Patients who refuse to continue to cooperate with the trial for various reasons;
4. Patients with obvious delayed awakening or unable to complete intubation in the operating room after the operation.

### 2.3 Randomization and blinding

Randomization is performed by an independent statistician and a random sequence is generated using SAS V.9.4 software (SAS Institute, Cary, NC) the numbers stored in opaque envelopes. Patients who meet the criteria will be randomly divided into treatment group and control group in a 1:1 ratio. The anesthesiologist, the attending physician, the patient, the associate investigator, the statistical analyst, and the nurse will be blinded to the assignment. To maintain blinding, dexmedetomidine hydrochloride for the treatment group and 0.9% sodium chloride solution for the control group will be in the same package and appearance.

### 2.4 Anesthesia scheme

After entering the operating room, the patients are connected to the ECG monitor and monitored the blood oxygen saturation. Venous access is established, and catheterization through the radial artery and the internal jugular vein is performed to measure the pressure and monitor the bispect ral index. All intravenous general anesthesia are used. Anesthesia induction consists intravenous injection of midazolam 0.02∼0.04 mg/kg, etomidate 0.1∼0.3 mg/kg, sufentanil citrate 0.2∼0.3 μg/kg, cisatracurium besylate 0.3 mg/kg, endotracheal intubation under video laryngoscopic and mechanical ventilation with a ventilator. Intravenous anesthesia is maintained with propofol and remifentanil, and 0.05∼0.20 μg/kg/min of remifentanil is continuously pumped with a microinfusion pump, and sufentanil citrate and atracurium cis-benzenesulfonate are added intermittently according to the operation conditions. The intraoperative fluctuation of blood pressure is kept within 10% of the baseline value. The intraoperative oxygen saturation is maintained >98%, and the target value of bispect ral index is maintained at 45 to 60. 5∼10 μg sufentanil and 50 mg flurbiprofen are given for preanalgesia half an hour before the end of operation. After surgery, the patient is not given antagonistic muscle relaxants. The endotracheal catheter is removed when the patient resumed spontaneous respiration, tidal volume >6 mL /kg, respiratory rate <30 times/min, PETCO_2_ maintained at 35 to 45 mmHg (1 mmHg = 0.133 kPa), and the patient can open his eyes and clench his hand. Patient-controlled intravenous analgesia, drug formulation for ketorolac tromethamine 240 mg, tropane SiQiong 5 mg, 0.9% sodium chloride solution diluted to 100 mL, infusion rate of 2 mL/h, patient-controlled analgesia volume of 0.5 mL, locking time for 15 minutes, maintaining postoperative pain in patients with Visual analogue scale ≤3.

In the treatment group, dexmedetomidine hydrochloride (Hengrui Pharmaceuticals, Jiangsu China, Batch No. 181112BP) is injected with a microinfusion pump within 15 minutes before anesthesia induction with preinjection loading dose (0.5 μg/kg), followed by continuous infusion at a rate of 0.3 μg/kg/h (dexmedetomidine hydrochloride is prepared with 0.9% sodium chloride solution to 4 μg/mL).

Whereas in the control group, patients will be continuously pumped with 0.9% sodium chloride solution at the corresponding pumping rate until the end of surgical anesthesia.

### 2.5 Outcome measures

1. Incidence of POD: the incidence of postoperative delirium in all patients will be evaluated at 1, 3, and 7 days after operation^[15]^ at the same time, and the incidence of postoperative delirium will be calculated, according to the four characteristics of delirium.
2. Cognitive status score: mini-mental state examination will be used to evaluate the cognitive function of the patients on the 1st day before operation, 1st day, 3rd day and 7th day after operation.
3. Adverse reactions, and the postoperative adverse reactions related to anesthesia are recorded.

### 2.6 Data collection and management

The data in this study will be collected by 2 assistants and entered into a predesigned table. The relevant information and data of this study will be collected, shared, and stored in a separate storeroom to protect the confidentiality before, during, and after the test. People outside this research group do not have access to relevant data. Without the written permission of the participants, the research information of the participants will not be published outside the study.

### 2.7 Statistical analysis

The data will be analyzed by SPSS 22.0 (SPSS Inc, Chicago, IL), with double-tail test, and the α level is set at *0.05*. The measurement data in accordance with normal distribution and homogeneity of variance will be tested by independent sample *t* test, and those who do not match will be analyzed by Mann–Whitney *U* test.

## 3 Discussion

The mechanism of delirium is not clear, which may be related to inflammation,^[16]^ sleep deprivation, physiological stress, traumatic stimulation, drugs (anticholinergic, opioid, benzodiazepine drugs),^[17]^ and neurological damage caused by cerebral hypoxia.^[18]^ Surgery can cause stress response, release inflammatory mediators, and induce delirium.^[19]^

Dexmetomidine is a selective receptor agonist. Its main target is located in the locus ceruleus of the human brainstem. It produces an analgesic effect by activating the locus ceruleus, spinal cord, and peripheral a-2 receptors of the central nervous system. At the same time, it causes a sedative effect similar to physiological sleep. It has the characteristics of fast metabolism and short action time.^[20]^ Experimental studies have found that dexmedetomidine can inhibit inflammatory factors through NF-B pathway,^[21]^ protect hippocampal neuronal HT22 cells from apoptosis induced by anesthetics by promoting PI3K/AKT pathway and inhibiting HIF-PKM2 axis,^[22]^ and enhance the anti-inflammatory properties of cholinergic pathway through a-7 cholinergic receptors.^[23]^ Clinical studies have shown that dexmedetomidine can reduce the use of opioids and benzodiazepines, reduce the risk of delirium caused by opioids and benzodiazepines, and protect patients’ sleep.^[24]^ At present, the efficacy of dexmedetomidine has been verified in cardiac surgery, but there are few clinical studies on long-term laparotomy in elderly patients. Therefore, we designed this randomized controlled study to determine the efficacy of dexmetomidine in reducing the incidence of postoperative delirium compared with placebo.

In addition, there are still some shortcomings in the design of this study. First of all, the diseases included for laparotomy are not limited, which may lead to some bias. Second, this study is a single-center study, the source of the case is relatively single and limited, which may affect the conclusions of the study to some extent.

## Author contributions

**Data curation:** Xiaoyuan Sui and Qingmei Duan.

**Funding acquisition:** Cuicui Li.

**Investigation:** Kunling Liu.

**Resources:** Kunling Liu and Cuicui Li.

**Software operating:** Qingmei Duan and Kunling Liu.

**Supervision:** Qingmei Duan.

**Writing – original draft:** Xiaoyuan Sui and Qingmei Duan.

**Writing – review & editing:** Xiaoyuan Sui, Cuicui Li.
